# Coping Strategies among Urban Poor: Evidence from Nairobi, Kenya

**DOI:** 10.1371/journal.pone.0083428

**Published:** 2014-01-10

**Authors:** Djesika D. Amendah, Steven Buigut, Shukri Mohamed

**Affiliations:** 1 African Population and Health Research Center, Nairobi, Kenya; 2 Department of Accounting, Finance and Economics, American University in Dubai, Dubai, United Arab Emirates; University of Oxford, Kenya

## Abstract

**Aims:**

In Kenya, it is estimated that 60 to 80% of urban residents live in slum or slum-like conditions. This study investigates expenditures patterns of slum dwellers in Nairobi, their coping strategies and the determinants of those coping strategies.

**Method:**

We use a dataset from the Indicator Development for Surveillance of Urban Emergencies (IDSUE) research study conducted in four Nairobi slums from April 2012 to September 2012. The dataset includes information related to household livelihoods, earned incomes of household members, expenditures, shocks, and coping strategies.

**Results:**

Food spending is the single most important component, accounting for 52% of total households' income and 42% of total expenditures. Households report a variety of coping strategies over the last four weeks preceding the interview. The most frequently used strategy is related to reduction in food consumption, followed by the use of credit, with 69% and 52% of households reporting using these strategies respectively. A substantial proportion of households also report removing children from school to manage spending shortfalls. Formal employment, owning a business, rent-free housing, belonging to the two top tiers of income brackets, and being a member of social safety net reduced the likelihood of using any coping strategy. Exposure to shocks and larger number of children under 15 years increased the probability of using a coping strategy.

**Policy Implications:**

Policies that contain food price inflation, improve decent-paying job opportunities for the urban poor are likely to reduce the use of negative coping strategies by providing urban slum dwellers with steady and reliable sources of income. In addition, enhancing access to free primary schooling in the slums would help limit the need to use detrimental strategies like “removing” children from school.

## Introduction

Over the past few decades, a rapid urbanization fueled by migration from rural to urban areas, occurred in sub-Saharan Africa countries concomitantly with slow economic growth. The proportion of Africans living in urban areas increased from 15% in 1950 to 39% in 2010 and is expected to rise to 43% in 2020[1]. Central and local government were unable to provide public services to a growing proportion of urban residents who ended up living in informal settlements characterized by high unemployment rate, poor housing, and poor public service provision including health, education and security [2]. According to 2006 estimates [3], up to 72% of urban dwellers in most sub-Saharan Africa countries (SSA) live in slums. In Kenya for instance, it is estimated that 60 to 80% of urban residents live in slum or slum like conditions [3].

In Nairobi, the capital city of Kenya, 73% of slum residents fell below the poverty line calculated using an expenditure-based poverty line of KES 3,174 (USD 37.7) per adult excluding rent per month in 2006 [4]. In addition, non-monetary indicators of well-being show great deprivation. For instance, housing units in the slums are mainly made of iron sheets providing inadequate protection from heat or cold. Nairobi informal settlements are also known to have worse health indicators than the national or the city average. A series of censuses that the African Population and Health Research Center (APHRC) have conducted in two Nairobi informal settlements (Korogocho and Viwandani) from 2006 indicate that in 2012, only 6% of households had access to piped water [5] and 51% shared toilets [6]. In addition, in these slums, three households out of four have no garbage disposal arrangement. In comparison, in 2008 (last national data available) 33% of households in urban areas had access to piped water in their dwelling and 52% to shared sanitation facility [7]. These national proportions are expected to improve with time so that corresponding proportions in 2012 will probably indicate a bigger gap in water and sanitation conditions between the slums areas and the rest of Nairobi. As a consequence of inadequate sanitation, open spaces, a nearby river and drains are used as garbage disposal and toilets in the slums, leaving residents vulnerable to diseases caused by poor personal hygiene, water and sanitation conditions. The poor living conditions contribute to higher mortality in the slums. Under five mortality rate was 86.2 in the slums compared to 63.5 for Nairobi and 74.5 for urban Kenya average in 2008 (last available data) [5]. Maternal mortality rate in two Nairobi slums was computed to be 706 per 100 000 live births [8], 30% higher than the national average [7] in 2008. Moreover, lack of security and violence are a serious problem and slum households are more likely to be subject to crime [4], [9].

Despite the growing and projected numerical importance of slum dwellers in Kenya and SSA, and the documented poor living conditions in the slum, most research on poverty, shocks and coping strategies focuses on rural areas [10]. While acknowledging the existence of a few studies relating to urban poverty in SSA, and in Kenya [11]–[13], a dearth of knowledge exists on the financial circumstances of slum dwellers, and their coping strategies. The current study seeks to investigate expenditure patterns, coping strategies and their determinants among residents of informal settlement in Nairobi living in the context of poverty. The results might provide useful insights for policy making concerning urban poor.

The remaining parts of the paper are structured as follows: Section 2 briefly discusses the literature on shocks, and coping strategies in Africa and in urban slums. Section 3 describes the study setting, data collection procedures and methods of data analysis. Section 4 presents the results and Section 5 concludes.

## Brief Literature Review

Households are subject to two types of shocks or adverse events: covariate shocks that affect the whole community and idiosyncratic shocks that affect a particular household or individual. While covariate and idiosyncratic shocks have a substantial impact on both urban and rural households' vulnerability, idiosyncratic shocks have a relatively higher impact on urban households' vulnerability or probability to fall into poverty [14]. The literature indicates that while households use various risk-coping strategies, those are not equally accessible to all. For instance, poorer households may be less able to use mechanisms that rely on prior savings, or assets as collateral [15]. Shocks and their coping mechanisms may generate poverty and/or cause its persistence [14] through the destruction or the reduction of the production capital of the household or by a negative behavioral change. Indeed, a household affected by a shock may change its behavior, by subsequently choosing low risk activities and asset portfolios resulting in lower mean returns and incomes. Thus, a better understanding of shocks and coping mechanisms may provide useful insights in designing poverty reduction policies.

Coping strategies can be divided into *ex-ante* and *ex-post* strategies [16]. Ex-ante strategies are protective risk-management actions by households before an eventual shock. These strategies usually take the form of insurance; self-insurance like precautionary savings and assets accumulation or community-based formal or informal insurance. Several studies have documented substantial heterogeneity in household saving behavior [17]. E*x-post* strategies are actions taken by households to mitigate the consequences of an adverse event. Example of these strategies are reducing expenditures, increasing home production or diversifying sources increasing of income [18]. Such strategies may have short-term or long-term impacts. Usually, households first implement coping mechanisms with short-term effect such as using up savings or selling assets, and when those mechanisms fall short, households may resort to other strategies with more long term effects such as withdrawing children from schools [19].

A review of the Kenya Integrated Household Budget Survey (2005-6) suggests that the most common coping strategies at the national level rank from spending cash saving, selling animals, to working longer hours, reducing food consumption and receiving help from family and friends [20]. A study in 15 lower middle and low income SSA countries indicated that borrowing and selling assets are common strategies adopted to cope with uninsured catastrophic health expenditures [21]. Households with higher inpatient expenditure and lower income were found to be more likely to borrow and sell assets.

Based on the above literature, we examine households' ex-ante coping strategies by looking at their ability to meet monthly expenditures and their savings then their ex-post coping strategies and its determinants.

## Methods

### Study setting

This paper used a dataset from the Indicator Development for Surveillance of Urban Emergencies (IDSUE) research study, funded by United States Agency for International Development's (USAID) Office of U.S Foreign Disaster Assistance (OFDA). The African Population and Health Research Center (APHRC) in partnership with Concern Worldwide run the project with the aim of developing early warning indicators to identify slow-onset humanitarian emergencies in urban slums. The study was conducted first in two Nairobi slums—Korogocho, Viwandani— for the first three data collection rounds before being extended to two additional Nairobi slums—Mukuru and Dandora from round 4, and two slums in Kisumu, another Kenya city from round 5. The four Nairobi sites were chosen based on logistical feasibility. Korogocho and Viwandani are study sites where the APHRC had been conducting the Nairobi Urban Health and Demographic Surveillance System (NUHDSS) since 2003. The NUHDSS is a framework of census data collected routinely among households living in the Demographic Surveillance Area. The NUHDSS collects every four months vital events such as births, deaths, migration in households that live within the area. This framework provides the basis for other nested studies.

### Sampling methods and field operations

The quantitative data collection for this IDSUE project begun in March 2011 and by January 2013 the study had collected five rounds of data. In each of the rounds, households were randomly selected. The first three rounds of data collection (round 1 to round 3) involved only Korogocho and Viwandani. Mukuru and Dandora slums which are very close in proximity to the HDSS sites were added in the fourth round. The study collected data using a household level survey conducted through an interviewer-administered questionnaire. The data were collected among residents, with residency defined as a minimum continuous stay of three months. In rounds 1 to 3, households were randomly selected from the NUHDSS database and interviewed. In round 4 and 5 with the inclusion of two other informal settlements where no enumeration area listings of the population existed, the selection of the households was modified. Households were randomly selected using a modified cluster sampling based on segmentation of villages. Each village in the slums was further broken down into segments of approximately equal size and the segments were all numbered. A random sample of the segments was taken and from each of the selected segments, all the households were listed and a random sample of the households to interview was taken from each selected segment. The fieldworkers collected and recorded the survey information on either a netbook or a mobile phone system, while the team leaders planned the survey collection logistics, managed the data collection, observed interviews, and checked the data for quality and consistency.

We used the datasets for the 4 Nairobi slums (i.e. Viwandani, Korogocho, Dandora and Mukuru and for rounds 4 and 5 for which the same sampling methods and questionnaires were utilized.

### Ethical consideration

In all selected households, the head of the household (or his/her representative) was first approached to obtain consent to the household participating in the household interview and signed a pre-written consent form. All participants who consented to participate confirmed this by signing a written consent form. A resident respondent who is knowledgeable about the household finances and other affairs was then interviewed. Ethical approval for the IDSUE study was obtained from the Kenya Medical Research Institute (KEMRI) national ethical review committee.

APHRC and Concern Worldwide co-own the IDSUE data. APHRC has data sharing policy under which data collected by the center is accessible to other researchers. More information is available at http://www.aphrc.org/images/Downloads/data%20sharing%20policy.pdf

### Data collection

The questionnaire used for rounds 4 and 5 included detailed expenditures and income questions for all the household members. In addition, the questionnaire include information related to food security, water and sanitation, household livelihoods, coping strategies, personal and property security, and food and non-food consumption, household expenditures and main breadwinner's income among others. Data collection for rounds 4 and 5 occurred between April-May 2012 and August-September 2012 respectively. Expenditure and income data were collected in Kenya Shillings (KES). The average exchange rate during the survey period (April 2012 to September 2012) was calculated to be 84.2 KES for 1USD.

We took specific measures to collect income data as our respondents work mainly in the informal sector. Income data were collected by asking the respondent first the number of household members who earned income, their age, gender and source of income. Then we asked the amount earned during the last payment period, and the regularity of the earnings.

In addition, for the general quality of the data, field interviewers were organized in team headed by a supervisor. The supervisor is the first to check the quality of the data at the end of each day, scrutinising missing, skips, values entered etc. Then the supervisor conducts spot checking on a few questions of each module of 5% of the questionnaires selected randomly 5%. At the first stage of data cleaning, high incomes were checked. Supervisors went back to households with total earned income higher than a pre-set value to verify information collected.

### Main variables

The questionnaire collected the income of the household members who earned income in the last 4 weeks. Daily or weekly income was adjusted to a monthly value with the appropriate multiplier. We aggregated the earnings of all workers to obtain the household income. This household income was divided into tertiles. The expenditure data collection was item specific with appropriate reference period. When reference periods were different, expenditures were all consolidated to represent the same unit of four weeks. For instance, utilities expenditures reference period was the last month before the interview while that of other clothing items were the three months before the interview to account for the fact that utility bills are monthly and clothing pieces are bought on a less regular basis. In that case, we divided the clothing expenditure by 3. Special care was taken to collect accurate information on food. First, details of food consumption for the previous day were collected, and then the total food expenditures information was collected for the 7 days prior to the interview. For this article, the weekly expenditure on food was extrapolated to four weeks.

We focus on seven coping strategies with the reference period of the last four weeks prior to the interview. Those coping strategies relate to whether a household member(s) (1) ate fewer number of meals per day due to lack of food (2) spent a whole day without eating due to lack of food (3) purchased household goods on credit (4) took a loan to buy food and other essentials (5) removed a child from school (6) left due to lack of resources (7) begged for food or money. We also constructed a binary variable = 1 if the household reported using any of the seven coping strategies. The survey did not ask whether the respondents used any of the coping strategies specifically after a shock.

Information on the coping strategies and the demographic variables such as sex, age of the main highest earner of the household, then number of children under the age of 15, were collected from the respondent.

### Data analysis

We first provide descriptive statistics of the general characteristics of the sample, and a summary of the distribution of expenditures, income and savings. We then describe the ex-post coping strategies used by households in the slums. Finally, we run a logistic regression for each coping strategy and also for the use of any of the seven. We present the odds-ratio of the determinants of the use of any coping strategy and for each of the seven strategies identified. We used Stata 12 (State College) for the analysis.

## Results

### Descriptive statistics

Table 1 indicates that proportions of the respondents from each of the four informal settlements are similar ranging from 24 to 27%. The majority of households surveyed are renters as only a small proportion (13%) of them live in rent-free dwellings. Among the households surveyed, 15% are migrants from rural areas and the average length of stay in the informal settlement is 8 years. In about two-thirds (66%) of the households the main bread winner is a male.

**Table 1:** Descriptive statistics of variables included in the analysis.

Variables	N	Description	Mean (standard deviation)	Min (max)
Data collection Round	3435	round 4	0.47 (0.50)	0 (1)
		round 5	0.53 (0.50)	0 (1)
Slum*	3435	Viwandani	0.25 (0.43)	0 (1)
		Korogocho	0.27 (0.44)	0 (1)
		Dandora	0.24 (0.42)	0 (1)
		Mukuru	0.25 (0.43)	0 (1)
Gender of main breadwinner	3435	Male = 1	0.64 (0.48)	0 (1)
		Female = 0	0.46 (0.48)	0 (1)
Tenure	3435	If household owns housing or housed free of charge = 1	0.13 (0.33)	0 (1)
		Otherwise = 0	0.87 (0.33)	0 (1)
Migrant from rural area	3435	If household is originally from rural area = 1	0.08 (0.26)	0 (1)
		Otherwise = 0	0.92 (0.26)	0 (1)
Years in slum	3435	Number of years household has stayed in the village	7.99 (8.62)	0 (58)
Childunder15	3435	No child under 15 = 1	0.12 (0.33)	0(1)
		Between 1 and 2 children under 15 = 1	0.69 (0.46)	0(1)
		More than 2 children under 15 = 1	0.18 (0.38)	0(1)
Age in years	3337	Age of main income earner	33.83 (9.97)	16 (80)
Main source of livelihood	3435	If has formal labor = 1	0.18 (0.38)	0 (1)
		If has own business = 1	0.16 (0.36)	0 (1)
		Otherwiseα = 0	0.67 (0.47)	0 (1)
Income group	3435	Lower third income group = 1;	0.36 (0.48)	0 (1)
		Middle third income group = 1;	0.31 (0.46)	0 (1)
		Top third income group = 1	0.33 (0.47)	0 (1)
Safety net	3435	If a member of household is enrolled in a social safety net e.g. merry-go-round = 1	0.26 (0.44)	0 (1)
		Otherwise = 0	0.74 (0.44)	0 (1)
Shocks	3435	if household experienced shockβ = 1	0.15 (0.35)	0 (1)
		Otherwise = 0	0.90 (0.35)	
Idiosyncratic shocksβ	3435	If household experienced idiosyncratic shocksγ = 1	0.10 (0.30)	0 (1)
		Otherwise = 0	0.90 (0.30)	0 (1)
Covariate shocksγ	3435	If household experienced communal/covariate shock ∼ Floods = 1	0.05 (0.21)	0 (1)
		Otherwise = 0	0.95 (0.21)	0 (1)

• The total may not add to 1 because of rounding errors.

do not have formal employment nor own a business. Household engaged in casual work, petty trade or is unemployed.

Idiosyncratic shock included : fire, mugging, burglary, eviction, property destruction, or rape.

Covariate shocks is floods.

The main income earner in the households is about 34 years old. In terms of the source of livelihood, fewer than one in four (18%) actually have a formal employment operationalized as a regular employment with a steady payment. A slightly smaller proportion (16%) owned a business. The larger majority (67%) do not have a steady source of income as they are engaged in casual work, petty trade or are unemployed.

Members of about 26% of households are involved in some kind of safety net such as a merry-go-round. In the last month before the survey, 15% of the households experienced some shocks, mostly idiosyncratic (10%), such as burglary, fire, floods, mugging, eviction, property destruction or rape etc. Only flood was considered as covariate shock and 5% of the households in the sample were affected in the last 4 weeks before the interview. Note that almost all shocks analyzed here involve an income or property loss. Five persons have declared having been raped.

### Distribution of income and expenditures

The average mean expenditure is KES 13957.1 (with a standard deviation of 10,009.33). Food spending is the single most important component and it absorbs on average 52% of the total household income and 42% of total expenditures (Table 2). Education cost (schooling of children) is a distant second consuming to 13% of total household income and accounting for 10% of the total household expenditure. Rent is the third with 12% and 9% of total income and expenditures respectively. Other significant elements are energy—10% of total income and 8% of total expenditure—transport and household basic items (tying at 9% of total household income and 7% of total expenditures).

**Table 2:** Mean and percentage of total household monthly income and expenditures per category.

Variable	Mean monthly Expenditure	Standard deviation	% of total household incomeα	% of total household expenditureβ
Foodµ	5,892	3,075	52	42
Education	1,425	4,238	13	10
Rent	1,298	1,203	12	9
Energy	1075	738	10	8
Transport	1,002	1,893	9	7
Basic household items	1,027	1,122	9	7
Clothing	408	666	4	3
Medical	323	3,428	3	2
Electricity	225	390	2	2

Mean household income 11,274 (standard deviation 9,417) and median household income is KES 8,800 (or about USD 104.5 per month).

Mean household expenditures: KES 13,957 (standard deviation 10,009).

Number of observations  = 3431 for food and 3435 for all other items.

### Coping strategies used by households

**Figure 1:**
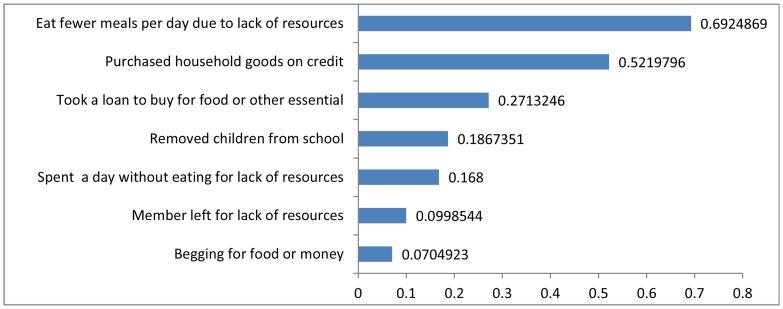
Percentage of households that used listed coping strategies in the last four weeks.

Each household interviewed was asked a series of questions on coping strategies used in the last four weeks. Specifically, the question was “during the last four weeks, did you or a family member” have to eat fewer meals, go a whole day without eating, took a loan to buy food etc. Households reported using various copying strategies in the four weeks preceding the interview. The main ones are indicated in Figure 1. The most frequently used strategy is related to reduction in food consumption: more than two-third of the households (69%) report eating fewer numbers of meals during a day. The second most commonly used strategy is accessing credit: more than half the households (52%) report purchasing household goods or food on credit. Loan facility is used by about 27% of the households. It is worth noting that about one household out of five (19%) report removing children from school to manage spending shortfalls. Also 42% of households use more than one coping strategies (not shown). On the other hand, only 21% of the households report using no copying strategy in the four weeks prior to the survey.

### Determinants of use of specific coping strategy

Table 3 provides results from several regression analyses with the dependent variables being the use of any or a specific coping strategy. The first column analyses the determinants of use of any coping strategy by the household. The following seven columns analyze the determinants of the other specific coping strategies covered by the household survey.

**Table 3:** Odds-ratios and (standard deviation) for the determinants of use of coping Strategies.

Determinant variables	Dependent variable = 1 if the household reported using the coping strategy							
	any coping strategy (1)	Access to credit		Reduced food consumption		Other		
		Purchased household goods on credit (2)	Taking a loan to buy food and other essentials (3)	Household member(s) spending a whole day without eating due to lack of food (4)	Household member(s) ate fewer number of meals per day due to lack of food (5)	Household member begging for food or money (6)	Household member had to leave for lack of resources (7)	Household remove children from school (8)
Formal labor	0.45 (0.05)***	0.51 (0.05)***	0.52 (0.06)***	0.43 (0.07)***	0.40 (0.04)***	0.30 (0.08)***	0.61 (0.11)***	0.52 (0.09)***
Own business	0.46 (0.06)***	0.53 (0.06)***	0.48 (0.06)***	0.40 (0.07)***	0.43 (0.05)***	0.28 (0.08)***	0.47 0.10***	0.54 (0.09)***
Tenure	0.62 (0.09)***	0.65 (0.08)***	0.85 (0.11)	0.90 (0.14)	0.56 (0.07)***	1.19 (0.26)	0.70 (0.15)	0.65 (0.11)**
Migrant from rural area	0.98 (0.20)	1.01 (0.15)	1.38 (0.21)**	0.85 (0.16)	0.79 (0.13)	0.94 (0.25)	0.79 (0.20)	1.32 (0.27)
One or two children under <15	1.15 (0.17)	1.39 (0.17)***	1.08 (0.14)	1.03 (0.16)	1.28 (0.17)*	1.00 (0.20)	1.10 (0.19)	1.30 (0.32)
More than 2 children under 15	1.37 (0.27)	1.68 (0.25)***	1.29 (0.21)	1.18 (0.23)	1.66 (0.29)***	0.92 (0.24)	0.81 (0.19)	2.12 (0.55)***
Main income earner is male	0.93 (0.10)	1.05 (0.09)	1.06 (0.10)	0.71 (0.08)***	0.92 (0.09)	0.75 (0.11)*	0.87 (0.11)	1.21 (0.15)
Safety net	0.70 (0.07)***	0.84 (0.07)**	1.14 (0.11)	0.54 (0.07)***	0.63 (0.06)***	0.88 (0.15)	0.87 (0.12)	0.87 (0.11)
Idiosyncratic shocksβ	2.76 (0.57)***	1.45 (0.18)***	1.64 (0.21)***	1.58 (0.24)***	1.74 (0.27)***	1.26 (0.27)	2.22 (0.37)***	2.14 0.35)***
Community shocksγ	3.62 (1.10)***	1.90 (0.36)***	2.71 (0.48)***	1.99 (0.41)***	3.16 (0.76)***	3.50 (0.84)***	2.70 (0.60)***	1.78 (0.41)**
Income group (middle one third)	0.59 (0.07)***	0.66 (0.06)***	0.73 (0.07)***	0.55 (0.07)***	0.58 (0.06)***	0.44 (0.08)***	0.74 (0.11)**	0.70 (0.09)***
Income group (top one third)	0.43 (0.05)***	0.52 (0.05)***	0.56 (0.06)***	0.40 (0.05)***	0.49 (0.05)***	0.32 (0.06)***	0.51 (0.08)***	0.47 (0.07)***
Years in slum	1.01 (0.01)	1.00 (0.01)	1.00 (0.01)	1.00 (0.01)	1.01 (0.01)	1.01 (0.01)	1.01 (0.01)	1.00 (0.01)
Age of main income earner	1.01 (0.01)	1.01 (0.00)***	1.01 (0.00)*	1.01 (0.01)**	1.01 (0.01)***	1.01 (0.01)	1.01 (0.01)**	1.03 (0.01)***
Round 5	0.97^a^ (0.09)^b^	0.88 (0.07)	0.84 (0.07)**	0.46 (0.05)***	0.91 (0.08)	1.07 (0.16)	1.12 (0.16)	0.75 (0.09)**
Korogocho	4.11 (0.60)***	1.09 (0.12)	1.36 (0.17)**	2.58 (0.46)***	4.23 (0.54)***	1.23 (0.33)	0.91 (0.18)	1.03 (0.19)
Mukuru	3.43 (0.48)***	1.40 (0.16)***	1.68 (0.22)***	4.12 (0.75)***	4.57 (0.59)***	2.82 (0.73)***	1.24 (0.25)	3.90 (0.70)***
Dandora	2.50 (0.35)***	2.15 (0.25)***	2.25 (0.30)***	3.36 (0.63)***	2.89 (0.36)***	3.95 (1.00)***	0.47 (4.81)***	2.36 (0.45)***
Constant	2.78 (0.76)***	0.69 (0.15)*	0.24 (0.06)***	0.16 (0.05)***	1.04 (0.25)	0.06 (0.02)***	0.07 (0.02)***	0.05 (0.02)***
Number Observations	3,337	3,337	3,337	3,337	3,336	3,335	3,337	2,462

Note: The second column shows the determinants of use of any coping strategies, the next seven analyze the use of specific coping strategies.

*is significant at 10%, ** significant at 5%, and *** significant at 1%.

Idiosyncratic shocks includes fire, mugging, burglary, eviction, property destruction or rape.

community shocks includes floods.

The determinants of the use of the various coping strategies seem relatively consistent across the specific strategies. Formal employment, owning a business, belonging to the two top tiers of income brackets, and being a member of social safety net clearly reduce the likelihood of using any coping strategy. For instance, households when the main income earner is in the reference category (casual work, petty trade or unemployment) have 2.5 (1/0.40) times higher odds of eating fewer meals a day due to lack of food than households with main earner in the formal labor category. Both formal employment and owning a business variables are found to reduce the likelihood of use of any of the seven specific copying strategies significantly (p<0.001). In the same vein, higher income decreases the probability of using any coping strategy. Relative to lower income group (low one third reference category), households in the middle income level have a decreased probability of using coping strategies. The level of statistical significance varies from 1% to 10% depending on the strategy. For instance, households in the lower third income group have 1.7 (1/0.59) times higher odds of using any coping strategy as households in the middle third. The magnitude of the protective effect of income is larger 2.3 (1/0.43) for the top level of income and statistically significant at 1% for all coping strategies. As for the availability of a social safety net such as merry-go-round to a family member, it reduces the probability that the household uses any coping strategy, but the magnitude of the effect is stronger and measured with precision with reduced food consumption strategies. Households whose member(s) belong to a safety group are 1.6 (1/0.63) less likely not to eat for a day and 1.9 (1/0.54) times more likely to eating fewer meals. Being a migrant from a rural appears to have a significant impact only on the likelihood of taking a loan to buy food.

On the other hand, the number of children under 15 years in the household increases the likelihood that the households “purchases household goods on credit”, “eat fewer meals” and “remove children from school”. The effect is stronger and statistically more significant for those with more than two children under 15 years. For instance, the odds that a household with more than two children remove children from school is 2.12 times as high as the odds in a family with no child under the age of 15 (p>0.01). The similar odds are 1.3 and not statistically significant for families with one or two children under the age of 15.

As expected, household level shocks and communal level shocks increase significantly the probability of use of a coping strategy. An intriguing result is that the older the main income earner, the more likely the use of a coping strategy. Although the magnitude of this effect is small, it is measured with precision for most coping strategies.

The variable ‘round’ was included to control for potential seasonality in using the strategies. Round 5 is significant for a few coping strategies including removing children from school, taking a loan and household members spending a whole day without food. Compared to Viwandani, the residents of the other informal settlements are more likely to use each one of the coping strategies.

## Discussion

This study found that food expenses represent 52% of the household's total income, and 40% of its total expenditures. Children education and rent respectively come second and third as components of household expenditures. The most common coping strategies to adverse events are reducing food consumption, use of credit and removing children from school. Having a formal employment or owning a business, belonging to the top third income bracket, and owning the dwelling appear to protect against the use of the most common negative coping strategies. On the other hand, having more than two children under 15 years, and having weathered a recent shock make the use of a negative coping strategy more likely.

The high share of food in the household income and expenditure is consistent with households living in poverty and has been document before [4], [22]. Indeed, the share of food in the household budget decreases with rising income according to Engel law [23]. Also, the importance of food in the household income suggests that slum dwellers are highly vulnerable to food price inflation. The Nairobi lower income annual inflation rate was 15% in 2011 compared to 10% for upper income due to the sharp increase in oil and food prices according to the Kenya National Bureau of Statistics [24]. Sensitivity of urban poor to food price inflation was documented in Ethiopia as well [25]. The high share of household income spent on food fits with the fact that the most common coping strategy used by household is reduced number of meals. This reduced food intake probably applies to all household members and may be related to the high level of stunting observed in the slums: 40% of children under the age of 5 are stunted in Korogocho and Viwandani slums [13].

Buying household items on credit is the second most common coping strategy. It also suggests that those households probably borrow (obtain goods on credit) from the “corner store” and they probably buy in small quantity and/or increment. A previous study indicated that items are more expensive in the slums as households tend to buy in very small quantities [4] and hence do not benefit from economies of scale.

The fact that households engaged in formal employment and those owning a business are less likely to use any of the coping strategies probably reflects the protective effect of a stable livelihood. In addition, higher income categories also protect against using any of the coping strategy. The effect of a higher and steady source of income suggests that policies that improve formal employment opportunities among the slums residents would contribute to reducing the use of negative coping strategy. This is even more important as the number of new jobs created in the informal sector in Nairobi is higher than the ones in the public and private sectors combined [24].

Households that own their houses or do not pay for it are also less likely to use a coping strategy. This variable features as significant in four of seven specific strategies. Owning or not paying rent has an income effect, freeing about 12% of the average household income that would have gone to the rent, to other necessities.

In addition, having more than two children under the age of 15 seems to increase the probability of using all the various coping strategies. The effect is especially large for the coping strategy of “removing children from school”. These higher probabilities would be as a result of the related burden of school costs and an increase in the dependency ratio inside the household. Studies focusing on education in Kenya found that about half the children in the slums of Korogocho and Viwandani do not benefit from the free primary education implemented in Kenya since 2003 as the “poorest of the poor” actually attend “private schools for the poor” in the absence of government schools in the slums [26]. In addition, even when primary school is accessible and “free”, other school-related costs such as textbooks, lunches, and uniforms represent a significant cost for the low income households. Private schools in the slum are fee-based and when parents owe tuition or other fees, children are sent home till the bill is cleared. So the “removing children from school” strategy may be temporary. But still, regular absenteeism is not conducive to good learning. Conventional wisdom indicates that income is positively related to level of education even in in these slums. Hence efforts to ensure children from slum families' access education may generate long term benefits and break the cycle of poverty. Thus, provision of free primary education for the slum families is an issue that needs serious consideration.

Access to social safety net such as merry-go-round mainly decreases the use of reduced food consumption as a coping strategy. This finding suggests that knowing and having the trust of other community members is a rampart against hunger. As expected households affected by any type of shock had increased probability of using coping strategies suggesting their vulnerability beforehand.

The paper presents two main limitations: The first one is that questionnaire did not ask specifically whether coping strategies were used in response to a specific shock. Thus we cannot establish whether the strategies are ex-post response to a specific adverse event or whether they are an answer to the underlying chronic poverty. Second, the paper is based on cross-sectional data. Thus, no causality can be ascertained. Panel datasets would have better helped explain the use of the coping strategies over time and the impact of shocks and other determinants on the use of those strategies.

## Conclusion and Policy Implications

This study found that food consumes more than half of the households' total income. Households' most common coping strategies adopted include: reducing food consumption, accessing credit and removing children from school. Having a formal job or owning a business or having relatively higher income appear to be protective factors against those negative coping strategies. Thus policies that result in reduced food price inflation in urban areas, improving decently paying job opportunities for the urban poor are likely to reduce the use of negative coping strategies by providing urban dwellers with steady and reliable source of income. For instance, excluding or reducing taxes on essential food items commonly used by the poor can help. In addition, implementation of free primary school in the slums is necessary to free parental income and most importantly reduce the recourse to “removing children from school” coping strategy that may have adverse long term effect. Free primary school policy in the slums may involve not only opening enough public schools for the population in there but also covering other school-related levees such as uniforms, exercise books, and lunch programs.
